# How information and communication overload impairs creative performance in the digital technology age: the chain mediating roles of emotion and state self-control

**DOI:** 10.3389/fpsyg.2026.1887168

**Published:** 2026-08-12

**Authors:** Meng Yang, Siyu Chen, Conghui Liu

**Affiliations:** Department of Psychology, Renmin University of China, Beijing, China

**Keywords:** creative performance, information and communication overload, negative affect, positive affect, state self-control

## Abstract

**Introduction:**

Despite the growing integration of digital technologies into work place innovation, information and communication overload in the age of pervasive digital devices remains under-researched regarding its impact on employee creativity. The present research examines the psychological mechanisms linking information and communication overload to creative performance.

**Methods:**

We conducted two multi-method studies to capture both stable interpersonal differences and dynamic intrapersonal fluctuations: Study 1 employed across-sectional design (*n* = 612), and Study 2 utilized a daily diary design (*n* = 66 across 10 consecutive workdays).

**Results:**

The results revealed that information and communication overload Significantly impairs creativity through both emotional and self-regulatory pathways. Furthermore, the findings revealed a dual-pathway chain mediation: information and communication overload exhausts an individual’s state self-control by reducing positive affect and increasing negative affect, both of which subsequently stifle creative output. Notably, where as the independent mediating role of negative affect was significant in Study 1, it was not supported in Study 2; however, the chain mediation pathway through negative affect and state self-control remained significant across both studies.

**Discussion:**

These findings deepen our understanding of how information and communication overload harms creative performance by highlighting self-regulation as a key mechanism. Practically, the results suggest that organizations must prioritize emotional replenishment and cognitive energy preservation to sustain creative capital in an increasingly saturated digital environment.

## 1 Introduction

In contemporary business environments, organizational competitive advantage is increasingly predicated on workforce creativity and innovation capacity (Anderson et al., 2014; Gehani, 2011). At the same time, the widespread adoption of digital technologies has fundamentally reshaped employees’ work contexts and cognitive processes, thereby influencing their creative performance (Tu et al., 2025). Creative performance, defined as the generation of novel, useful, and valuable ideas, methods, or products (Tierney et al., 1999), is a dynamic process involving problem identification, information processing, and solution evaluation (Green et al., 2023). To sustain innovation in rapidly evolving markets, organizations have increasingly embedded digital technologies into multiple dimensions of human resource management, including recruitment, employee development, and performance evaluation (Huang, 2025).

However, digital technology represents a double-edged sword. On the one hand, it can alleviate cognitive burdens through the automation of routine tasks and provide diverse informational inputs that stimulate idea generation (Dell'Acqua et al., 2026). On the other hand, excessive reliance on digital technologies may constrain cognitive flexibility, reduce creative self-efficacy, and ultimately suppress innovative output (Carroll and Conboy, 2020; Delpechitre et al., 2018; Dey et al., 2020; Karr-Wisniewski and Lu, 2010; Molino et al., 2020; Singh et al., 2022; Yu et al., 2018; Zhou et al., 2024). Moreover, employees’ emotional reactions to digital technologies may differ substantially across individuals, further complicating the net effect on creativity (Pittino et al., 2025). These contrasting effects raise an important question: whether the overuse of digital technologies—manifested as technology-related overload—may undermine employees’ creative performance.

Information and communication overload are two dimensions of technology-related overload (Karr-Wisniewski and Lu, 2010). Information overload occurs when individuals are exposed to volumes of data that exceed their cognitive processing capacity, whereas communication overload arises when employees face excessive interaction demands across multiple digital channels (e.g., email and instant messaging platforms). Research has linked these forms of overload to a range of negative workplace outcomes, including work–family conflict (Harris et al., 2015) and decreased job performance (Delpechitre et al., 2018; Karr-Wisniewski and Lu, 2010; Yu et al., 2018). Oldham and Da Silva (2015) argued that excessive use of digital technology elevates stress levels, which in turn undermines creative performance. Consistent with the Stressor–Strain–Outcomes (SSO) model (Koeske and Koeske, 1993), information and communication overload can be conceptualized as stressors that influence work outcomes through psychological strains (Byron et al., 2010; LePine et al., 2005). Accordingly, the first objective of this study is to examine the impact of information and communication overload on employees’ creative performance.

Additionally, it is also important to understand the underlying mechanisms through which information and communication overload influences creativity. Drawing on Gu et al. (2018) dual-process framework, which conceptualizes creativity as arising from both emotional and cognitive processes, this study investigates potential mediating pathways from both perspectives. Prior research on information and communication overload has predominantly focused on its effects on negative affect (e.g., Moqbel and Bartelt, 2018; Salanova et al., 2013), while largely overlooking the role of positive affect. To address this gap, the present study incorporates both positive and negative affect as mediating variables.

Besides, this study considers state self-control as a key cognitive mediator. State self-control refers to the momentary capacity to regulate thoughts, emotions, and behaviors in response to situational demands (de Ridder et al., 2018). As a limited cognitive resource, state self-control is highly susceptible to contextual influences (VanDellen and Hoyle, 2010), and prior research has demonstrated its relevance to creative performance (Chiu et al., 2017; Taylor, 2021). Therefore, positive affect, negative affect, and state self-control are jointly examined as mediating variables in the relationship between information and communication overload and creativity.

Finally, most existing studies on the negative consequences of information and communication overload have relied on cross-sectional designs (Chandra et al., 2019; Delpechitre et al., 2018; La Torre et al., 2020). To address this limitation, the present research employs both cross-sectional and longitudinal approaches. Specifically, Study 1 uses a cross-sectional design to test the proposed relationships, while Study 2 adopts a daily diary design to examine whether the observed associations replicate at the within-person level over time.

## 2 Theoretical background and hypotheses

### 2.1 Technology overload and creative performance

Information and communication technologies in contemporary workplaces have enhanced organizational connectivity and information accessibility. However, a growing body of research indicates that the excessive use of these tools may generate unintended negative consequences. As organizations increasingly undergo digital-intelligence transformation, employees face novel demands that may reshape their work behaviors and psychological states (Wu et al., 2025). Prior studies have shown that heavy reliance on digital communication systems can lead to workplace inefficiencies, including time fragmentation, workflow disruption, and attentional switching costs (Spira and Feintuch, 2005; Tams et al., 2020; Tandon et al., 2021). While these systems are designed to improve coordination efficiency, they may paradoxically increase perceived work pressure by raising expectations for immediacy and responsiveness (Wang et al., 2008). Research suggests that information and communication overload is closely associated with technostress-related outcomes such as reduced productivity and emotional exhaustion (Delpechitre et al., 2018; Karr-Wisniewski and Lu, 2010; Ragu-Nathan et al., 2008; Tiwari, 2020; Yu et al., 2018). Oldham and Da Silva (2015) suggested that employees using new technologies may spend too much time gathering information, resulting in an overabundance of ideas that cannot be effectively filtered and integrated, thereby reducing creativity. Additionally, stress resulting from information and communication overload may further damage creative performance (Liu and Liu, 2020; Oldham and Da Silva (2015); Talbot et al., 1992). Therefore, we propose the following hypothesis:

*H1*: Information and communication overload has a negative impact on creative performance.

### 2.2 The mediating role of emotion

Information and communication overload not only affects work-related behavior but also induces psychological changes, such as job dissatisfaction (Tarafdar et al., 2010) and fatigue (Salanova et al., 2013). Employees experiencing information and communication overload may feel anxious (Moqbel and Bartelt, 2018), and prolonged exposure can lead to negative affect, including depression and anger (Swar et al., 2017). Moreover, as a stressor, information and communication overload can disrupts the regulation of positive affect (Gilbert, 2012; Yang et al., 2018) and exerts considerable influence on individuals’ psychological wellbeing (Choi and Lim, 2016; Han and Huang, 2025; Matthes et al., 2020; Nimrod, 2017; Reinke and Ohly, 2021; Singh et al., 2022). Emotion is vital to employees’ creative performance. Previous research has demonstrated that positive affect and emotional engagement can enhance creativity (Carmeli et al., 2014; Conner and Silvia, 2015; Lapeniene and Dumciene, 2014; Zenasni and Lubart, 2002), and two meta-analyses have consistently shown that positive affect significantly facilitates creativity (Baas et al., 2008; Davis, 2009). In work settings, Madrid et al. (2014) found, through a longitudinal study, that positive affect positively predicts innovative work behavior. In contrast, negative affect impairs creativity (Isen et al., 1985, 1987; Wang and Jiang, 2022) by narrowing cognitive scope (De Dreu et al., 2008). According to Yeh et al. (2015), stress diminishes creativity by eliciting negative affect such as frustration and anger. Thus, we propose the following hypotheses:

*H2a*: Positive affect mediates the relationship between information and communication overload and creative performance.

*H2b*: Negative affect mediates the relationship between information and communication overload and creative performance.

### 2.3 The mediating role of state self-control

Information and communication overload can lead to exhaustion and concentration difficulties, making it challenging for employees to focus on their work (Ayyagari et al., 2011; Cao and Sun, 2018; Ragu-Nathan et al., 2008; Sheng et al., 2022). Chronic exposure to stressful situations also results in diminished self-control and reduced self-efficacy (Duckworth et al., 2013; Zhou et al., 2024). Coping with stress may necessitate suppressing thoughts and affects and managing attention, processes that consume self-control resources (Gailliot et al., 2007; Ren et al., 2010). When employees encounter novel and disruptive technological events, such as human–robot collaboration, their cognitive resources may be further strained, leading to heightened job insecurity and altered work behaviors (Wu et al., 2025). According to the strength model, self-control constitutes a finite resource, and its depletion leads to impaired performance on subsequent self-control tasks (Hagger et al., 2010). Low state self-control has been associated with reduced creativity in most extant research (Price and Yates, 2015; Taylor, 2021). For instance, Taylor (2021) found that participants with low state self-control performed poorly on a creativity task requiring high levels of creative output. Similarly, depleted upper primary students exhibited lower creativity (Price and Yates, 2015). Moreover, self-control training can enhance creativity, as evidenced by improved insight problem-solving performance following self-control exercises (Chiu, 2014; Chiu et al., 2017). Therefore, we propose the following hypothesis:

*H3*: State self-control mediates the relationship between information and communication overload and creative performance.

### 2.4 The chain mediating role of emotion and self-control

Effects of different valences exert distinct effects on self-control. Positive affect can improve the level of self-control and reduce the impact of ego depletion (Baumeister, 2003; Gong and Li, 2017; Isen and Reeve, 2005; Tice et al., 2007). Positive affect have been found to facilitate self-control behavior (Shmueli and Prochaska, 2012) and the ability to focus on task (Isen and Reeve, 2005). Conversely, negative affect immerses individuals in aversive experiences, depletes limited psychological resources, and undermines self-control (Fu and Tremayne, 2021; Gong and Li, 2017; Heatherton and Wagner, 2011; Muraven and Baumeister, 2000). Liu et al. (2020) found that positive and negative affect were positively and negatively correlated with self-control, respectively. Heatherton and Wagner (2011) believed that negative affect is particularly destructive and can impair self-control through multiple pathways. An fMRI study examined the physiological mechanism underlying this effect revealed that negative affect causes self-control failure by over-consuming inhibitory regions of the prefrontal cortex (Chester et al., 2016). Based on these findings, both the decrease in positive affect and the increase in negative affect triggered by information and communication overload can reduce state self-control. Therefore, we propose following hypotheses:

*H4a*: Positive affect and state self-control sequentially mediate the relationship between information and communication overload and creative performance.

*H4b*: Negative affect and state self-control sequentially mediate the relationship between information and communication overload and creative performance.

**Figure 1:**
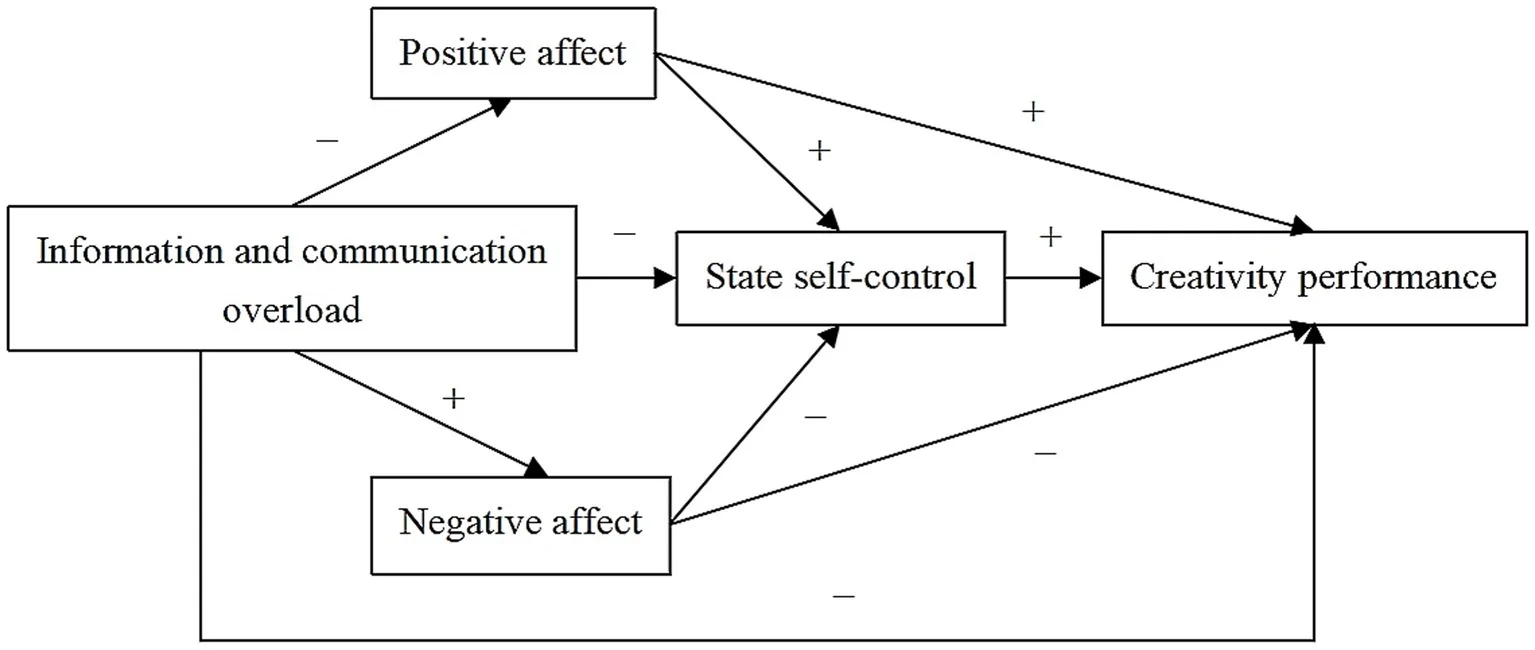
Hypothesized conceptual model. “+” refers to positive influence, “–” refers to negative influence.

Based on the four hypotheses outlined above, the hypothesized conceptual model is depicted in Figure 1.

## 3 Study 1

### 3.1 Methods

#### 3.1.1 Participants

A total of 722 employees who used information technology at work were recruited for this study using convenience and snowball sampling methods from various regions in the country. After excluding invalid data (e.g., submission within 2 min, and incorrect responses to the identification question, etc.), data from 612 participants were included in the final analysis. Two hundred and eight of the participants were male (34.0%). The majority (83.5%) of the participants were between 25 and 44 years old. The demographic characteristics of the final participants are presented in Supplementary Table 1.

#### 3.1.2 Measures

##### 3.1.2.1 Demographic information Questionnaire

We used a self-developed demographic questionnaire consisting of 9 items. The items included gender, age, educational background, marital status, children’s status, occupation, job level, years of work experience and monthly income level.

##### 3.1.2.2 Information and Communication Overload Scale

Information and communication overload was assessed with 7 items adapted from Karr-Wisniewski and Lu (2010) (e.g., “I am often distracted by the excessive amount of information available to me for business decision making.”). The scale comprises two sub-dimensions: information overload (3 items) and communication overload (4 items). Items were rated on a 5-point Likert-type scale ranging from 1 (*strongly disagree*) to 5 (*strongly agree*). The Cronbach’s *α* for this scale was 0.86 in the present study.

##### 3.1.2.3 Positive Affect and Negative Affect Scale

Positive and negative affect were assessed using the adapted PANAS developed by Qiu et al. (2008), which contains 9 positive adjectives (e.g., *active*, *enthusiastic*) and 9 negative ones (e.g., *upset*, *afraid*). Items were rated on a 5-point Likert-type scale ranging from 1 (*very slightly or not at all*) to 5 (*extremely*). The Cronbach’s α for the scale was 0.89 in this research.

##### 3.1.2.4 State Self-Control Scale

State self-control was assessed using the 10-item short version of the State Self-Control Capacity Scale (SSCCS), consisting of items 3, 6, 9, 10, 11, 12, 13, 15, 16, and 18 (Ciarocco et al., 2007). This scale measures individuals’ perceived momentary availability of self-control resources. Items were rated on a 7-point Likert-type scale ranging from 1 *(strongly disagree)* to 7 *(strongly agree)*. Eight of the 10 items are negatively worded and were reverse-scored (e.g., “I feel mentally exhausted”). After reverse-coding, a higher total score indicates a higher level of state self-control. The Cronbach’s α for this scale was 0.83 in the present study.

##### 3.1.2.5 Creativity job performance

Creative performance was assessed with 9 items adapted from Janssen and Van Yperen (2004) scale (e.g., “I am good at creating new ideas for improvements at work.”). The scale comprises three sub-dimensions: idea generation (3 items), idea promotion (3 items), and idea realization (3 items). Items were rated on a 6-point Likert-type scale ranging from 1 (*strongly disagree*) to 6 (*strongly agree*). The Cronbach’s α for the scale was 0.92 in the study.

#### 3.1.3 Procedure and analytical strategy

In mid-January 2022, we released our questionnaires onto the Sojump platform^1^ to collect participants’ recent status and experiences at work. All participants signed a consent form before they took the survey. They were informed that their data would be kept strictly confidential and only used for research purpose. After completing the questionnaire, participants were rewarded with a small amount of money. All procedures performed in studies involving human participants were in accordance with the ethical standards of the institutional and national research committee and with the 1964 Helsinki Declaration and its subsequent amendments.

After the data were collected, we examined common method variance with Harman’s single-factor analysis, and computed Pearson correlations to examine the relationships between the main variables using SPSS 28.0. We used Model 80 in Process 4.0 to analyze the mediating relationships among positive affect, negative affect, state self-control, and information and communication overload and creative performance. The hypothesized mediation model was tested using Model 80 of the PROCESS macro for SPSS (Hayes, 2018), which allows for parallel and serial mediation. Significance of the indirect effects was evaluated using bias-corrected percentile bootstrap confidence intervals based on 5,000 bootstrap samples. A confidence interval that does not include zero indicates a statistically significant indirect effect.

### 3.2 Results

#### 3.2.1 Confirmatory factor analysis

To evaluate the discriminant validity of the five focal constructs, we conducted a series of CFA comparing the hypothesized five-factor model with several alternative nested models. The hypothesized five-factor model demonstrated acceptable fit: χ^2^(892) = 2633.02, CFI = 0.89, TLI = 0.89, RMSEA = 0.06 [90% CI: 0.054, 0.059], SRMR = 0.08. Critically, the five-factor model fit the data significantly better than all alternative models.

Given that state self-control and negative affect may share conceptual overlap—particularly as the State Self-Control Capacity Scale includes items referring to feeling “drained” or “mentally exhausted”—we paid specific attention to models that merged these two constructs. The four-factor model merging state self-control with negative affect exhibited substantially worse fit [ΔCFI =−0.08, Δχ^2^(4) = 592.22, *p* < 0.001] (Supplementary Table 2). Standardized factor loadings for the five-factor model ranged from 0.51 to 0.85 (*ps* < 0.001). Discriminant validity was further assessed using the Fornell-Larcker criterion. The square root of the AVE for each construct exceeded its correlations with all other constructs.

To assess common method bias, we performed the Harman’s single-factor test to examine the potential common method variance. Results showed that 11 factors had eigenvalues higher than one. The first factor accounted for 20.4% of the variance, which was less than the recommended critical value of 40% (Zhou and Long, 2004). Therefore, common method variance was not significant in this study.

#### 3.2.2 Descriptive statistics and correlation analysis

The descriptive statistics and Pearson correlations for all study variables are presented in Table 1. Creative performance was negatively correlated with information and communication overload and negative affect, and positively correlated with positive affect and state self-control. Information and communication overload was positively associated with negative affect and negatively associated with positive affect and state self-control. Positive affect was positively related to state self-control, whereas negative affect was negatively related to state self-control.

**Table 1:** Descriptive statistics and Pearson correlations between variables in Study 1 (*n* = 612).

Variable	*M*	*SD*	1	2	3	4
1. Information and communication overload	3.37	0.76				
2. Positive affect	3.25	0.87	−0.18^***^			
3. Negative affect	2.21	0.91	0.18^***^	0.07		
4. State self-control	3.99	0.92	−0.43^***^	0.29^***^	−0.45^***^	
5. Creative performance	4.35	0.84	−0.15^***^	0.52^***^	−0.14^***^	0.36^***^

^***^p < 0.001.

#### 3.2.3 Mediation analysis

**Figure 2:**
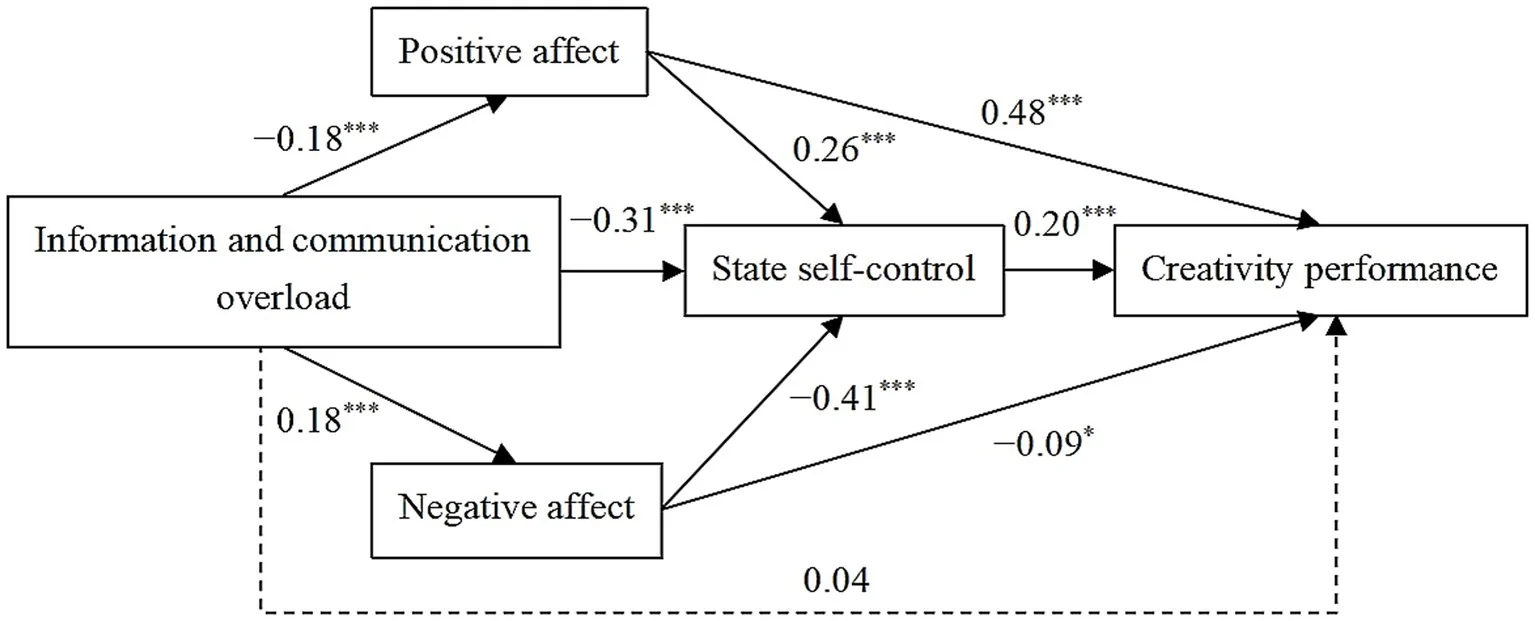
Final model with standardized coefficient for each path in Study 1. ^*^p < 0.05, ^***^p < 0.001.

As shown in Table 2, information and communication overload negatively predicted positive affect (*β* = −0.18, *SE* = 0.04, 95% CI [−0.26, −0.10]) and state self-control (*β* = −0.31, *SE* = 0.04, 95% CI [−0.39, −0.22]), and positively predicted negative affect (*β* = 0.18, *SE* = 0.04, 95% CI [0.09, 0.26]). Positive affect positively predicted state self-control (*β* = 0.26, *SE* = 0.04, 95% CI [0.19, 0.33]) and creative performance (*β* = 0.48, *SE* = 0.04, 95% CI [0.39, 0.56]). Negative affect negatively predicted state self-control (*β* = −0.41, *SE* = 0.04, 95% CI [−0.48, −0.34]) and creative performance (*β* = −0.09, *SE* = 0.04, 95% CI [−0.17, −0.01]). State self-control positively predicted creative performance (*β* = 0.20, *SE* = 0.04, 95% CI [0.12, 0.30]). Standardized coefficients for each path are presented in Figure 2.

**Table 2:** Descriptive statistics and Pearson correlations between variables in Study 1.

Model pathways	β	*SE*	95% CI
Regression path coefficients			
ICO → PA	−0.18	0.04	[−0.26, −0.10]
ICO → NA	0.18	0.04	[0.09, 0.26]
ICO → SSC	−0.31	0.04	[−0.39, −0.22]
PA → SSC	0.26	0.04	[0.19, 0.33]
NA → SSC	−0.41	0.04	[−0.48, −0.34]
ICO → CP	0.04	0.04	[−0.03, 0.12]
PA → CP	0.48	0.04	[0.39, 0.56]
NA → CP	−0.09	0.04	[−0.17, −0.01]
SSC → CP	0.20	0.04	[0.12, 0.30]
Direct effect	0.04	0.04	[−0.03, 0.12]
Indirect effect	−0.19	0.03	[−0.25, −0.13]
ICO → PA → CP	−0.09	0.02	[−0.13, −0.05]
ICO → NA → CP	−0.02	0.01	[−0.04, −0.003]
ICO → SSC → CP	−0.06	0.02	[−0.10, −0.03]
ICO → PA → SSC → CP	−0.01	0.004	[−0.02, −0.004]
ICO → NA → SSC → CP	−0.02	0.01	[−0.03, −0.01]

ICO, Information and communication overload; PA, Positive affect; CP, Creative performance; NA, Negative affect; SSC, State self-control; CI, bias-corrected percentile bootstrap confidence interval (5,000 resamples).

The direct effect of information and communication overload on creative performance was not significant (*β* = 0.04, *SE* = 0.04, 95% CI [−0.03, 0.12]). The total indirect effect was significant (*β* = −0.19, *SE* = 0.03, 95% CI [−0.25, −0.13]). As reported in Table 2. The indirect effect via positive affect was significant (*β* = −0.09, SE = 0.02, 95% CI [−0.13, −0.05]), supporting Hypothesis 2a. The indirect effect via negative affect was significant (*β* = −0.02, SE = 0.01, 95% CI [−0.04, −0.003]), supporting Hypothesis 2b. The indirect effect via state self-control was significant (*β* = −0.06, SE = 0.02, 95% CI [−0.10, −0.03]), supporting Hypothesis 3. The chain mediating effect of positive affect and state self-control was significant (*β* = −0.01, 95% CI [−0.02, −0.004]), supporting Hypothesis 4a. The chain mediating effect of negative affect and state self-control was also significant (*β* = −0.02, 95% CI [−0.03, −0.01]), supporting Hypothesis 4b. The total effect model explained 33.8% of the variance in creative performance.

We further examined whether information overload and communication overload exerted differential effects by estimating separate models for each dimension. The results revealed a largely consistent pattern across the two dimensions. Both information overload and communication overload exhibited significant negative total indirect effects on creative performance, with no significant direct effect in either model, confirming full mediation for both dimensions. The associations with positive affect, negative affect, and self-control were comparable in magnitude and direction, and the downstream pathways from affect and self-control to creative performance remained stable. Moreover, these dimensional findings aligned closely with the results obtained from the composite information and communication overload construct reported above. Detailed regression coefficients and indirect effects for both dimensional models are presented in Supplementary Table 3.

## 4 Study 2

### 4.1 Methods

#### 4.1.1 Participants

Participants in Study 2 were recruited on a voluntary basis from the pool of 612 respondents who had completed Study 1. During the Study 1 survey, participants were asked whether they would be willing to participate in a follow-up daily diary study; 134 employees provided informed consent and were enrolled. Due to a technical programming error in the online survey platform, the daily questionnaire link failed to be distributed correctly to all enrolled participants on the first 2 days of the study period, resulting in the loss of 28 participants who did not receive the survey. Of the remaining 106 participants who received the daily survey across the full 10-day period, we applied a compliance-based inclusion criterion consistent with established recommendations for intensive longitudinal designs: participants were retained if they completed the daily questionnaire on at least 8 of the 10 workdays (Gabriel et al., 2019; Ohly et al., 2010). Forty participants were excluded for failing to meet this criterion. The final analytic sample thus comprised 66 participants.

To evaluate the representativeness of the retained sample and assess potential attrition bias, we conducted two sets of comparative analyses. First, we compared the 66 retained participants with the 40 excluded participants on all demographic characteristics. There were no significant differences in gender distribution [χ^2^(1) = 0.13, *p* = 0.722], age [*t*(104) = 0.05, *p* = 0.959], job rank [*t*(104) = 0.66, *p* = 0.512], work tenure [*t*(104) = 0.55, *p* = 0.580], or monthly income [*t*(104) = 0.84, *p* = 0.403]. Second, we compared the 66 retained participants with the 40 excluded participants on the key variables. There were no significant differences in information and communication overload [*t*(104) = 0.03, *p* = 0.975], positive affect [*t*(104) = 0.43, *p* = 0.666], negative affect [*t*(104) = −0.22, *p* = 0.825], state self-control [*t*(104) = −1.67, *p* = 0.093], and creative performance [*t*(104) = 0.51, *p* = 0.610]. Participants completed an average of 9.81 days. The demographic characteristics of the final 66 participants are presented in Supplementary Table 3.

#### 4.1.2 Measures

##### 4.1.2.1 Daily Information and Communication Overload Scale

The Daily Information and Communication Overload Scale used the same questionnaire as in Study 1 and adapted it appropriately to include a time definer (e.g., “In today’s work, I am often distracted by the excessive amount of information available to me for business decision making.”). The scoring was consistent with the scale in Study 1. In this 10-day follow-up study, the mean Cronbach’s *α*for this scale was 0.90, ranging from 0.85 to 0.94.

##### 4.1.2.2 Daily PANAS

The Daily PANAS questionnaire was adapted from the PANAS questionnaire used in Study 1, with an instruction prompting participants to recall their emotional feelings at work today. The questions were also slightly modified by adding the word “today.” In this 10-day follow-up study, the mean Cronbach’s α for the scale was 0.85, ranging from 0.78 to 0.89.

##### 4.1.2.3 State Self-control Scale

Study 2 used the 5-item short version of the SSCCS, consisting of items 2, 6, 18, 19, and 21 (Ciarocco et al., 2007; Johnson et al., 2014). The instruction emphasized today’s work status. Items were rated on a 7-point Likert-type scale ranging from 1 *(strongly disagree)* to 7 *(strongly agree)*. All 5 items are negatively worded and were reverse-scored. A higher total or average score indicates a higher level of state self-control for that day. In this 10-day follow-up study, the mean Cronbach’s α for the scale was 0.93, ranging from 0.90 to 0.96.

##### 4.1.2.4 Daily Creativity Job Performance Scale

Daily creative performance was assessed with the Daily Creative performance Scale developed by Zhou and George (2001) with four items (e.g., Today I had new and creative ideas.). Items were answered on a 7-point Likert-type scale (1 = strongly disagree to 7 = strongly agree). In this 10-day follow-up study, the mean Cronbach’s α for the scale was 0.93, ranging from 0.87 to 0.95.

#### 4.1.3 Procedure and analytical strategy

The questionnaire survey was conducted over 10 consecutive working days, from January 17 to January 28, 2022. After obtaining participants’ consent, the questionnaire link was posted in the WeChat moment group at 16:00 each day, and participants were reminded to complete the questionnaire. The completion window was from 16:00 to 23:50 on each working day. To ensure data anonymity and confidentiality, participants were matched by IP address and WeChat authorized login credentials.

We performed data analyses using SPSS 28.0 and Mplus 8.3. Given the nested structure of the diary data, we employed multilevel modeling to account for the interdependence of repeated measurements. Prior to formal analysis, we assessed common method bias using the unmeasured latent methods factor approach (Podsakoff et al., 2003) and compared model fit between a five-factor model and a six-factor model. We then computed Pearson correlations to examine the bivariate relationships among the main variables at the between-person level. To test the hypothesized within-person indirect effects, we conducted multilevel path analysis in Mplus 8.3. Given the nested structure of the data, all Level 1 predictors were person-mean centered. We used the MODEL INDIRECT command to estimate the indirect effects. To obtain robust standard errors and confidence intervals that account for the non-normality often observed in indirect effects, we performed multilevel bootstrapping with 5,000 resamples. A 95% confidence interval that does not include zero indicates statistical significance.

### 4.2 Results

#### 4.2.1 Multilevel confirmatory factor analysis

Common method bias was tested by controlling for effects of an unmeasured latent methods factor (Podsakoff et al., 2003). Five variables in the study were used to establish a five-factor model, and the common method factor was added on the basis of the five-factor model. The fit indices of the six-factor model were χ^2^ = 1998.30, *df* = 1,021, *p* < 0.001, RMSEA = 0.04, SRMR (within individuals) = 0.04, SRMR (between individuals) = 0.05, CFI = 0.93, TLI = 0.92. Compared with the five-factor model, the change of each fit index was less than 0.01. Therefore, it can be considered that the model was not significantly improved after adding the common method factor. Therefore, there is no obvious common method bias in this study.

To address concerns regarding construct overlap, we conducted multilevel confirmatory factor analysis to examine the factor structure at both within-person and between-person levels. ICCs for the five focal variables ranged from 0.57 to 0.77, confirming substantial between-person variance and justifying the use of multilevel modeling.

We first specified a two-level five-factor model with identical factor structures at both within and between levels. The model demonstrated good fit: robust RMSEA = 0.05 [95% CI: 0.049, 0.051], SRMR (within) = 0.04, SRMR (between) = 0.07. To complement the multilevel CFA, we also conducted within-person level CFAs using person-mean centered data with cluster-robust standard errors. This approach isolates within-person fluctuations by removing stable between-person differences (Curran and Bauer, 2011). The within-person five-factor model demonstrated excellent fit: χ^2^(517) = 1034.20, CFI = 0.95, TLI = 0.94, RMSEA = 0.04 [95% CI: 0.036, 0.043], SRMR = 0.04. Standardized factor loadings ranged from 0.51 to 0.83 for all constructs (*ps* < 0.001) (Supplementary Table 4).

Critically, we compared the hypothesized five-factor model with several alternative nested models at the within-person level. The five-factor model fit significantly better than all alternatives. Importantly, the four-factor model merging state self-control with negative affect exhibited substantially worse fit: ΔCFI = −0.07, Δχ^2^(4) = 631.82, *p* < 0.001. To assess common method bias, we added an unmeasured latent method factor to the within-person five-factor model. The resulting changes in fit indices were all below the recommended threshold of 0.01 (Podsakoff et al., 2003), indicating that common method variance did not seriously threaten the validity of our findings.

#### 4.2.2 Descriptive statistics and correlation analysis

The descriptive statistics and Pearson correlations for all study variables are presented in Table 3. Information and communication overload was negatively correlated with positive affect, state self-control, and creative performance, and positively correlated with negative affect. The pattern of correlations was consistent with the results of Study 1, providing preliminary evidence for the hypothesized relationships.

**Table 3:** Descriptive statistics and Pearson correlations between variables in Study 2 (*n* = 66).

Variable	*M*	*SD*	1	2	3	4
1. Information and communication overload	3.13	0.89				
2. Positive affect	2.88	1.02	−0.36^***^			
3. Negative affect	1.89	1.04	0.51^***^	−0.33^***^		
4. State self-control	4.27	1.56	−0.68^***^	0.43^***^	−0.68^***^	
5. Creative performance	4.35	1.50	−0.49^***^	0.66^***^	−0.56^***^	0.56^***^

^***^p < 0.001.

#### 4.2.3 The mediation analyses

**Figure 3:**
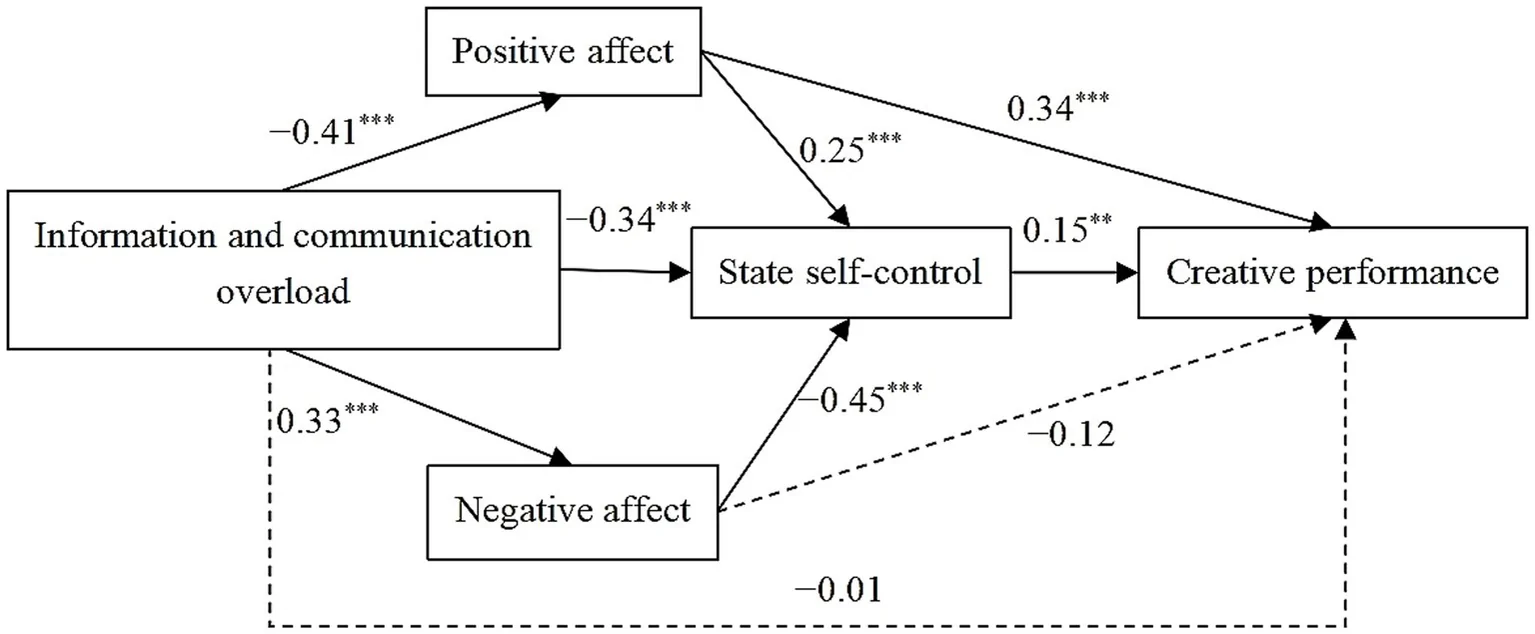
Final model with standardized coefficient at the within-individual level for each path in study 2. ^**^p < 0.01, ^***^p < 0.001.

We used the MODEL INDIRECT command to evaluate the significance of the mediating effect at the within-individual level. The results showed that the total effect of information and communication overload on creative performance was significant (*β* = −0.49, *SE* = 0.15, *t* = −3.34, *p* = 0.001). However, the direct effect of information and communication overload on creative performance was not significant (*β* = −0.02, *p* = 0.791). The indirect effect via positive affect was significant (*β* = −0.22, *SE* = 0.07, *t* = −3.16, *p* = 0.002), whereas the indirect effect via negative affect was not significant (*β* = −0.06, *p* = 0.277). The indirect effect via state self-control was significant (*β* = −0.10, *SE* = 0.03, *t* = −3.13, *p* = 0.002). The chain mediating effect of positive affect and state self-control was significant (*β* = −0.03, *SE* = 0.02, *t* = −2.03, *p* = 0.042), as was the chain mediating effect of negative affect and state self-control (*β* = −0.05, *SE* = 0.02, *t* = −2.56, *p* = 0.011). Overall, Hypotheses 1, 2a, 3, 4a, and 4b received support; Hypothesis 2b was not supported. Standardized coefficients for each path are shown in Figure 3. Detailed results, including path estimates and significance tests, are provided in Supplementary Tables 3–5.

As shown in Table 4, at the within-person level, information and communication overload negatively predicted positive affect (*β* = −0.41, *SE* = 0.04, 95% CI [−0.49, −0.32]) and state self-control (*β* = −0.34, *SE* = 0.04, 95% CI [−0.42, −0.25]), and positively predicted negative affect (*β* = 0.33, *SE* = 0.04, 95% CI [0.26, 0.41]). Positive affect positively predicted state self-control (*β* = 0.25, *SE* = 0.04, 95% CI [0.16, 0.34]) and creative performance (*β* = 0.34, *SE* = 0.05, 95% CI [0.25, 0.44]). Negative affect negatively predicted state self-control (*β* = −0.45, *SE* = 0.05, 95% CI [−0.56, −0.35]), but its direct effect on creative performance was not significant (*β* = −0.12, *SE* = 0.07, 95% CI [−0.25, 0.01]). State self-control positively predicted creative performance (*β* = 0.15, *SE* = 0.04, 95% CI [0.07, 0.24]). Standardized coefficients are presented in Figure 3.

**Table 4:** Standardized direct and indirect effect for the mediating model in study 2.

Model pathways	β	*SE*	95% CI
Regression path coefficients			
ICO → PA	−0.41	0.04	[−0.49, −0.32]
ICO → NA	0.33	0.04	[0.26, 0.41]
ICO → SSC	−0.34	0.04	[−0.42, −0.25]
PA → SSC	0.25	0.04	[0.16, 0.34]
NA → SSC	−0.45	0.05	[−0.56, −0.35]
ICO → CP	−0.01	0.04	[−0.09, 0.08]
PA → CP	0.34	0.05	[0.25, 0.44]
NA → CP	−0.12	0.07	[−0.25, 0.01]
SSC → CP	0.15	0.04	[0.07, 0.24]
Direct effect	−0.01	0.04	[−0.09, 0.08]
Indirect effect	−0.27	0.04	[−0.34, −0.20]
ICO → PA → CP	−0.14	0.02	[−0.20, −0.10]
ICO → NA → CP	−0.04	0.02	[−0.09, 0.004]
ICO → SSC → CP	−0.05	0.02	[−0.09, −0.02]
ICO → PA → SSC → CP	−0.01	0.01	[−0.03, −0.01]
ICO → NA → SSC → CP	−0.02	0.01	[−0.04, −0.01]

ICO, Information and communication overload; PA, Positive affect; CP, Creative performance; NA, Negative affect; SSC, State self-control; CI, bias-corrected percentile bootstrap confidence interval (5,000 resamples).

The direct effect of information and communication overload on creative performance was not significant (*β* = −0.01, *SE* = 0.04, 95% CI [−0.09, 0.08]). The total indirect effect was significant (*β* = −0.27, *SE* = 0.04, 95% CI [−0.34, −0.20]). As reported in Table 4, regarding specific indirect effects, the indirect effect via positive affect was significant (*β* = −0.14, SE = 0.02, 95% CI [−0.20, −0.10]), supporting Hypothesis 2a. In contrast to Study 1, the indirect effect via negative affect was not significant (*β* = −0.04, SE = 0.02, 95% CI [−0.09, 0.004]), failing to support Hypothesis 2b in the daily diary context. The indirect effect via state self-control was significant (*β* = −0.05, SE = 0.02, 95% CI [−0.09, −0.02]), supporting Hypothesis 3. The chain mediating effect of positive affect and state self-control was significant (*β* = −0.01, SE = 0.01, 95% CI [−0.03, −0.01]), supporting Hypothesis 4a. The chain mediating effect of negative affect and state self-control was also significant (*β* = −0.02, SE = 0.01, 95% CI [−0.04, −0.01]), supporting Hypothesis 4b. The model explained 29.5% of the within-person variance in creative performance.

We further tested whether technology overload on a given day affected creative performance on the following day through next-day affect and self-control. Lagged multilevel models were estimated using the lme4 package in R, with prior-day creative performance and study day included as covariates. Prior-day technology overload did not significantly predict next-day positive affect (*β* = −0.04, *p* = 0.377), negative affect (*β* = 0.03, *p* = 0.360), or state self-control (*β* = −0.08, *p* = 0.312). All lagged indirect effects were non-significant (via positive affect: −0.026; via negative affect: −0.020; via state self-control: −0.025). These results might suggest that the effects of technology overload on creative performance operate primarily within the same day rather than spilling over to the next day. Full results are reported in Supplementary Table 6.

## 5 Discussion

This paper used cross-sectional and longitudinal research methods to explore the mechanism by which information and communication overload weakens employee creative performance through emotion and state self-control. Consistent with our hypothesis, previous study (Luqman et al., 2021, Oldham and Da Silva, 2015) and the Stress–Strain-Outcome model (Koeske and Koeske, 1993), both studies 1 and 2 indicated negative and significant correlations between information and communication overload and creative performance. Moreover, Moreover, we found that the relationship between information and communication overload and creative performance was mediated by positive affect and state self-control, as well as through the chain mediation pathways of both positive affect–state self-control and negative affect–state self-control. These findings help us focus on how to reduce the indirect impact of information and communication overload on creative performance and adopt more targeted strategies.

Across both Study 1 and Study 2, positive affect significantly mediated the relationship between information and communication overload and creative performance, indicating that information and communication overload can impair creativity by diminishing positive affect. The relationship between information and communication overload and positive emotion is consistent with a recent study which found that perceived information overload caused by smartphone use is negatively correlated with positive emotion (Li and Chan, 2021). information and communication overload can cause anxiety and stress(Cao and Sun, 2018; Sheng et al., 2022), leading to a reduction in positive emotions(Schick et al., 2021). According to the Broaden-and-Build Theory of positive emotions (Fredrickson, 2001), positive emotion can enhance creativity by broadening attention and cognition scopes. This path is partly in line with Sriwidharmanely et al. (2022) study, which found that positive emotion raised by positive feedback can buffer the negative effect of technostress. It could be confirmed that positive emotion has an important mediating role between information and communication overload and work performance, including creativity.

The mediating effect of negative affect on the relationship between information and communication overload and creative performance produced inconsistent conclusions across Studies 1 and 2. In Study 1, the indirect effect via negative affect was significant, whereas in Study 2 this pathway was not supported. Such results might be due to the difference in measurement instruments. Study 1 employed a 9-item creative performance scale that captures idea generation, promotion, and realization (Janssen and Van Yperen, 2004), whereas Study 2 used a 4-item daily creativity scale focusing more narrowly on idea generation (Zhou and George, 2001). The broader scope of the Study 1 measure may be more sensitive to the detrimental effects of negative affect, particularly on the social and implementation aspects of creativity, which are not fully captured by the daily idea generation measure. Second, at the daily level, the influence of negative affect on creative performance may be more context-dependent than at the between-person level (Binnewies and Wörnlein, 2011; Bledow et al., 2013; To et al., 2012), potentially contingent on moderators such as emotion regulation strategies or psychological capital—variables not measured in the present study. Future research should employ consistent and comprehensive creativity measures across study designs to rule out measurement-related explanations for divergent findings.

State self-control played a significant mediating role in the relationship between information and communication overload and creative performance in both Study 1 and 2, indicating that information and communication overload might decrease state self-control and thus harm creativity. Information and communication overload could cause exhaustion and stress (Cao and Sun, 2018; Sheng et al., 2022), further leading to low self-control ability (Duckworth et al., 2013), which could explain the first stage of the path. For the second stage, a meta-analysis has showed that self-control is related to many domains, including school and work achievement (de Ridder et al., 2012). Moreover, because the psychological resources consumed by self-control are domain general, the consumption of resources in one domain will result in the decrease of self-control energy in other domains (Baumeister et al., 1998). This study extends the domain of self-control influence to creative performance.

The chain mediating role of affect and self-control between information and communication overload and creativity was also confirmed in both Study 1 and Study 2. Effects of different valences exert distinct effects on self-control. Negative affect can impair executive functions, which are necessary for self-control due to their top-down inhibition ability (Curci et al., 2013). Neuroimaging evidence shows that negative affect excessively recruit inhibitory regions of the prefrontal cortex, causing poor self-control (Chester et al., 2016). In contrast, positive affect can broaden attention scope and foster flexible thinking (Fredrickson, 2001), leading to effective self-control (Aspinwall, 1998; Trope and Neter, 1994). Consistent with Isen and Reeve (2005), we found that people experiencing positive affect showed better self-control and spend more time on work tasks rather than enjoyable tasks. Our chain mediating results are also consistent with the Stressor-Strain-Outcomes model (Koeske and Koeske, 1993) and previous research on creativity, which suggests that creativity is the result of a combination of factors, including affect, cognition, motivation, and external environmental factors (Niu, 2007; Shalley et al., 2004; Soroa et al., 2015).

This research makes several theoretical and practical contributions. First, we extend the literature by simultaneously examining positive affect, negative affect, and state self-control as parallel and sequential mediators linking information and communication overload to creative performance—a comprehensive dual-pathway model that prior research has not tested. Second, by combining a cross-sectional design with a longitudinal diary study, we provide more robust and convergent evidence than most existing studies (Chandra et al., 2019; Delpechitre et al., 2018; La Torre et al., 2020), which have relied predominantly on single-occasion surveys. The consistency of results across the two designs strengthens confidence in the proposed mediation model. Third, our findings carry practical implications for organizations that depend on technology to drive efficiency (Rasool et al., 2021). We underscore that information and communication overload is fundamentally an organizational stressor rather than merely an individual coping deficit, and mitigating its effects therefore requires systemic, organizational-level interventions. From a human resource management perspective, organizations should implement comprehensive information-flow governance, establish clear digital communication norms, reduce unnecessary meetings and instant-message interruptions, integrate fragmented digital tools, and simplify complex system functions. While emotional regulation training can serve as a supplementary resource, the primary focus must be on redesigning the digital work environment to address the structural sources of overload. By doing so, organizations can effectively preserve employees’ positive affect and state self-control, thereby enabling technology to deliver greater benefits and strengthening the enterprise’s innovation capacity.

### 5.1 Limitations

Several limitations should be noted. First, both studies relied on self-report questionnaires, which are susceptible to common method and social desirability biases. Future research should adopt multi-source designs, combining self-reports with supervisor ratings or objective indicators of creative output, and employ more precise measures that match the targeted construct. Second, the cross-sectional design of Study 1 precludes causal inferences, and the same-day measurement of all variables in Study 2 limits temporal separation, leaving reverse causality plausible. Future studies should adopt time-lagged or experience-sampling designs with multiple daily measurements to disentangle the temporal ordering of within-person processes. Third, while we screened for invalid responses, momentary emotional and self-control states remain vulnerable to interference from extraneous events. Fourth, although day-level measurement may overlook finer-grained dynamics, we opted for this approach because information overload is often a sustained experience with delayed effects on creativity, and frequent interruptions for event-level reporting could themselves induce overload (Sonnentag et al., 2018; Weinhardt and Carey, 2000). Future research could examine these relationships at a more refined temporal resolution. Despite these limitations, this study advances our understanding of how information and communication overload impairs creative performance and offers practical implications for fostering innovation in organizations.

## 6 Conclusion

We explored the effect of information and communication overload on employee creativity and the mediating role of emotion and state self-control, using both a cross-sectional study and a longitudinal diary study. Our findings revealed that information and communication overload indirectly and negatively predicted creative performance through positive affect and state self-control. Additionally, positive affect–state self-control and negative affect–state self-control played a chain mediating role in the relationship between information and communication overload and creative performance across both studies. Notably, the independent mediating role of negative affect was significant in Study 1 but not in Study 2. These findings extended the research in this field and have practical implications for enterprise innovation.

## Data Availability

The raw data supporting the conclusions of this article will be made available by the authors, without undue reservation.

## Footnotes

1
https://www.wjx.cn/
