# Supplementary material for: How information and communication overload impairs creative performance in the digital technology age: the chain mediating roles of emotion and state self-control
Source: Front Psychol. 2026 Aug 12;17:1887168. doi: 10.3389/fpsyg.2026.1887168 (PMC13506495; doi:10.3389/fpsyg.2026.1887168)
Supplement: Supplementary file 1 [file Table_1.docx]

Supplementary Material

# 1 Supplementary Tables

**Supplementary Table 1.** Sample Demographic Characteristics of Study 1 (*N*=612).

| **Demographic Variables** | **Categories** | **Frequency** | **Valid Percentage (%)** | **Cumulative Percentage (%)** |
| --- | --- | --- | --- | --- |
| Gender | Male | 208 | 34.0 | 34.0 |
|  | Female | 404 | 66.0 | 100.0 |
| Age | ≤ 24 years | 68 | 11.1 | 11.1 |
|  | 25–34 years | 378 | 61.8 | 72.9 |
|  | 35–44 years | 133 | 21.7 | 94.6 |
|  | 45–54 years | 29 | 4.7 | 99.3 |
|  | ≥ 55 years | 4 | 0.7 | 100.0 |
| Job Rank | Ordinary Staff | 305 | 49.8 | 49.8 |
|  | Frontline Manager | 184 | 30.1 | 79.9 |
|  | Middle Manager | 105 | 17.2 | 97.1 |
|  | Senior Manager | 18 | 2.9 | 100.0 |
| Work Tenure | ≤ 2 years | 153 | 25.0 | 25.0 |
|  | 2–5 years | 215 | 35.1 | 60.1 |
|  | 5–10 years | 152 | 24.8 | 85.0 |
|  | 10–20 years | 70 | 11.4 | 96.4 |
|  | ≥ 20 years | 22 | 3.6 | 100.0 |
| Monthly Income (CNY) | < 5000 | 116 | 19.0 | 19.0 |
|  | 5000–9999 | 277 | 45.3 | 64.2 |
|  | 10000–14999 | 111 | 18.1 | 82.4 |
|  | 15000–19999 | 53 | 8.7 | 91.0 |
|  | 20000–24999 | 18 | 2.9 | 94.0 |
|  | 25000–29999 | 18 | 2.9 | 96.9 |
|  | ≥ 30000 | 19 | 3.1 | 100.0 |

**Supplementary Table 2.** Confirmatory Factor Analysis Model Comparison of Study 1.

| **Model** | **χ²** | **df** | **CFI** | **TLI** | **RMSEA [90% CI]** | **SRMR** | **ΔCFI** | **Δχ²** |
| --- | --- | --- | --- | --- | --- | --- | --- | --- |
| 5-factor model | 2633.02 | 892 | 0.89 | 0.89 | 0.056  [0.054, 0.059] | 0.08 | — | — |
| 4-factor model A (SC + NA merged) | 3980.08 | 896 | 0.81 | 0.80 | 0.075  [0.073, 0.077] | 0.11 | 0.08 | 592.22***  Δ*df* = 4 |
| 4-factor model B (ICO + SC merged) | 3631.48 | 896 | 0.83 | 0.82 | 0.071  [0.068, 0.073] | 0.09 | 0.06 | 7422.67***  Δ*df* = 4 |
| 3-factor model (PA + NA + SC merged) | 8089.99 | 899 | 0.55 | 0.53 | 0.114  [0.112, 0.117] | 0.18 | 0.34 | 3033.62***  Δ*df* = 7 |
| 1-factor model | 11111.83 | 902 | 0.36 | 0.33 | 0.136  [0.134, 0.138] | 0.19 | 0.53 | 14458.39***  Δ*df* = 10 |

*Notes*. *N* = 612. ICO = information and communication overload; PA = positive affect; NA = negative affect; SC = state self-control; CP = creative performance. All ΔCFI values represent comparisons against the hypothesized five-factor model. Δχ² values are based on Satorra-Bentler scaled chi-square difference tests for the 5-factor versus each alternative model.

****p* < 0.001.

**Supplementary Table 3.** Dimensional Comparison: Information Overload vs. Communication Overload.

|  | **Information Overload** |  | **Communication Overload** |
| --- | --- | --- | --- |
| **Regression paths** | **β [95% CI]** |  | **β [95% CI]** |
| X → PA | −0.160 [−0.249, −0.080]*** |  | −0.171 [−0.256, −0.095]*** |
| X → NA | 0.198 [0.112, 0.279]*** |  | 0.143 [0.066, 0.233]** |
| X → SSC | −0.290 [−0.374, −0.215]*** |  | −0.276 [−0.351, −0.193]*** |
| PA → SSC | 0.268 [0.196, 0.344]*** |  | 0.268 [0.193, 0.339]*** |
| NA → SSC | −0.407 [−0.487, −0.334]*** |  | −0.425 [−0.493, −0.360]*** |
| PA → CP | 0.473 [0.392, 0.560]*** |  | 0.476 [0.393, 0.570]*** |
| NA → CP | −0.089 [−0.170, −0.006]* |  | −0.087 [−0.172, −0.009]* |
| SSC → CP | 0.192 [0.102, 0.279]*** |  | 0.206 [0.121, 0.299]*** |
| **Direct effect** | 0.015 [−0.064, 0.082] |  | 0.055 [−0.022, 0.129] |
| **Indirect effect** | −0.173 [−0.238, −0.121]*** |  | −0.173 [−0.235, −0.120]*** |
| X → PA → CP | −0.076 [−0.121, −0.039]*** |  | −0.082 [−0.127, −0.044]*** |
| X → NA → CP | −0.018 [−0.038, −0.002]* |  | −0.012 [−0.033, −0.002]* |
| X → SSC → CP | −0.056 [−0.091, −0.029]*** |  | −0.057 [−0.092, −0.031]*** |
| X → PA → SSC → CP | −0.008 [−0.017, −0.003]* |  | −0.009 [−0.019, −0.004]** |
| X → NA → SSC → CP | −0.016 [−0.029, −0.007]** |  | −0.013 [−0.025, −0.005]* |

*Notes*. X = Information overload (left) or Communication overload (right); PA = positive affect; NA = negative affect; SC = state self-control; CP = creative performance. CI = bias-corrected percentile bootstrap confidence interval (5,000 resamples).

*p* < 0.05. ** *p* < 0.01. *** *p* < 0.001.

**Supplementary Table 4.** Sample Demographic Characteristics of Study 2 (*N*=66).

| **Demographic Variables** | **Categories** | **Frequency** | **Valid Percentage (%)** | **Cumulative Percentage (%)** |
| --- | --- | --- | --- | --- |
| Gender | Male | 22 | 33.3 | 33.3 |
|  | Female | 44 | 66.7 | 100.0 |
| Age | ≤ 24 years | 5 | 7.6 | 7.6 |
|  | 25–34 years | 46 | 69.7 | 77.3 |
|  | 35–44 years | 9 | 13.6 | 90.9 |
|  | 45–54 years | 5 | 7.6 | 98.5 |
|  | ≥ 55 years | 1 | 1.5 | 100.0 |
| Job Rank | Ordinary Staff | 27 | 40.9 | 40.9 |
|  | Frontline Manager | 22 | 33.3 | 74.2 |
|  | Middle Manager | 16 | 24.2 | 98.5 |
|  | Senior Manager | 1 | 1.5 | 100.0 |
| Work Tenure | ≤ 2 years | 15 | 22.7 | 22.7 |
|  | 2–5 years | 18 | 27.3 | 50.0 |
|  | 5–10 years | 20 | 30.3 | 80.3 |
|  | 10–20 years | 8 | 12.1 | 92.4 |
|  | ≥ 20 years | 5 | 7.6 | 100.0 |

**Supplementary Table 5.** Within-person confirmatory factor analysis model comparison in Study 2.

| **Model** | **χ²** | **df** | **CFI** | **TLI** | **RMSEA [90% CI]** | **SRMR** | **ΔCFI** | **Δχ²** |
| --- | --- | --- | --- | --- | --- | --- | --- | --- |
| 5-factor model | 1034.20 | 517 | 0.95 | 0.94 | 0.039  [0.036, 0.043] | 0.04 | — | — |
| 4-factor model A (SC + NA merged) | 1703.86 | 521 | 0.87 | 0.86 | 0.059  [0.056, 0.062] | 0.05 | 0.07 | 631.82*** |
| 4-factor model B (ICO + SC merged) | 1560.22 | 521 | 0.89 | 0.88 | 0.055  [0.052, 0.059] | 0.05 | 0.06 | 198.73*** |
| 3-factor model (PA + NA + SC merged) | 2638.08 | 524 | 0.77 | 0.76 | 0.079  [0.076, 0.082] | 0.07 | 0.17 | 718.08*** |
| 1-factor model | 3724.31 | 527 | 0.66 | 0.64 | 0.097  [0.094, 0.100] | 0.09 | 0.29 | 893.49*** |

*Notes*. *N* = 648 observations, 66 participants. ICO = information and communication overload; PA = positive affect; NA = negative affect; SC = state self-control; CP = creative performance. Within-person CFA used person-mean centered data with cluster-robust standard errors (MLR estimator). ΔCFI values represent comparisons against the hypothesized five-factor model. Δχ² values are based on Satorra-Bentler scaled chi-square difference tests.

***p < .001.

**Supplementary Table 6.** Results of lagged multilevel Models: Prior-day information and communication overload predicting next-day mediators and creative performance

|  | **β** | ***SE*** | ***df*** | ***t*** | ***p*** |
| --- | --- | --- | --- | --- | --- |
| **Model 1a: ICO(t−1) → PA(t)** |  |  |  |  |  |
| Intercept | 0.000 | 0.023 | 579 | 0.000 | 1.000 |
| ICO(t−1) | −0.038 | 0.043 | 579 | −0.884 | 0.377 |
| Day | −0.010 | 0.009 | 579 | −1.122 | 0.262 |
| **Model 1b: PA(t) & ICO(t−1) → CP(t)** |  |  |  |  |  |
| Intercept | 0.018 | 0.159 | 65.1 | 0.112 | 0.911 |
| PA(t) | 0.670 | 0.054 | 512.1 | 12.385 | < 0.001 |
| ICO(t−1) | −0.008 | 0.059 | 512.1 | −0.132 | 0.895 |
| CP(t−1) | −0.007 | 0.040 | 512.1 | −0.178 | 0.859 |
| Day | 0.033 | 0.012 | 512.1 | 2.888 | 0.004 |
| **Model 2a: ICO(t−1) → NA(t)** |  |  |  |  |  |
| Intercept | 0.000 | 0.019 | 579 | 0.000 | 1.000 |
| ICO(t−1) | 0.033 | 0.036 | 579 | 0.916 | 0.360 |
| Day | 0.002 | 0.007 | 579 | 0.309 | 0.758 |
| **Model 2b: NA(t) & ICO(t−1) → CP(t)** |  |  |  |  |  |
| Intercept | 0.018 | 0.159 | 65.1 | 0.112 | 0.911 |
| NA(t) | −0.586 | 0.068 | 512.1 | −8.574 | < 0.001 |
| ICO(t−1) | −0.015 | 0.063 | 512.1 | −0.229 | 0.819 |
| CP(t−1) | −0.008 | 0.042 | 512.1 | −0.198 | 0.843 |
| Day | 0.028 | 0.012 | 512.1 | 2.282 | 0.023 |
| **Model 3a: ICO(t−1) → SSC(t)** |  |  |  |  |  |
| Intercept | 0.000 | 0.040 | 579 | 0.000 | 1.000 |
| ICO(t−1) | −0.076 | 0.075 | 579 | −1.011 | 0.312 |
| Day | −0.010 | 0.015 | 579 | −0.628 | 0.530 |
| **Model 3b: SSC(t) & ICO(t−1) → CP(t)** |  |  |  |  |  |
| Intercept | 0.018 | 0.159 | 65.1 | 0.112 | 0.911 |
| SSC(t) | 0.321 | 0.032 | 512.1 | 9.948 | < 0.001 |
| ICO(t−1) | 0.004 | 0.062 | 512.1 | 0.070 | 0.944 |
| CP(t−1) | 0.020 | 0.041 | 512.1 | 0.474 | 0.635 |
| Day | 0.029 | 0.012 | 512.1 | 2.435 | 0.015 |
| **Model 4: Full model (all mediators)** |  |  |  |  |  |
| Intercept | 0.018 | 0.159 | 65.1 | 0.112 | 0.911 |
| PA(t) | 0.489 | 0.063 | 510.1 | 7.701 | < 0.001 |
| NA(t) | −0.155 | 0.079 | 510.1 | −1.954 | 0.051 |
| SSC(t) | 0.134 | 0.040 | 510.1 | 3.379 | < 0.001 |
| ICO(t−1) | 0.001 | 0.058 | 510.1 | 0.015 | 0.988 |
| CP(t−1) | −0.006 | 0.039 | 510.1 | −0.165 | 0.869 |
| Day | 0.033 | 0.011 | 510.1 | 2.942 | 0.003 |

*Note*. N = 582 daily observations, 66 participants. ICO= information and communication overload; PA = positive affect; NA = negative affect; SSC = state self-control; CP = creative performance.
